# Immune checkpoint inhibitor (ICI) combination therapy compared to monotherapy in advanced solid cancer: A systematic review

**DOI:** 10.7150/jca.49174

**Published:** 2021-01-01

**Authors:** Xiaoting Ma, Yujian Zhang, Shan Wang, Huamin Wei, Jing Yu

**Affiliations:** 1 Cancer Center, Beijing Friendship Hospital, Capital Medical University, No. 95 Yong An Road, Xi Cheng District, Beijing, 100050, China.; 2 Department of Traditional Chinese Medicine, Beijing Friendship Hospital, Capital Medical University, No. 95 Yong An Road, Xi Cheng District, Beijing, 100050, China.

**Keywords:** ICI, combination, efficacy, malignancy, meta-analysis, safety

## Abstract

**Aim:** To evaluate the efficacy and safety of immune checkpoint inhibitor (ICI) two-drug combination therapy in patients with advanced malignancy.

**Methods:** We searched PubMed, PMC, EMBASE, EBSCO, Cochrane Central Register of Controlled Trials (CENTRAL), American Society of Clinical Oncology (ASCO and the European Society of Medical Oncology (ESMO) to identify primary research reporting the survival outcomes and safety of ICI combination therapy in patients with advanced malignancy. We performed a meta-analysis that evaluated the risk ratio (RR) and its 95% confidence interval (CI) for objective response rates (ORR) and disease control rates (DCR), hazard ratio (HR) and 95% CI for progression-free survival (PFS) and overall survival time (OS), and RR and 95% CI for adverse events (AEs).

**Results:** The final 10 studies (15 cohorts) and 2410 patients were included in the meta-analysis. The ICI combination therapy resulted in improved ORR (RR 1.82, 95% CI 1.31-2.54, *p* = 0.0004), DCR (RR 1.41, 95% CI 1.29-1.55, *p* < 0.0001), PFS (HR 0.83, 95% CI 0.74-0.94, *p* = 0.003) and OS (HR 0.90, 95% CI 0.82-0.98, *p* = 0.02) in patients with advanced malignant tumors. The incidence of some high grade (≥3) AEs increased, such as fatigue, nausea, diarrhea, colitis, rash, pruritus, elevated transaminase and lipase.

**Conclusion:** Our study showed that ICI combination therapy can improve ORR, DCR, PFS and OS in patients with advanced malignancy. Compared with ICI monotherapy, ICI combination therapy was more likely to induce severe AEs.

## Introduction

In recent years, with the in-depth study of tumor immunology, immune checkpoint inhibitors, CAR-T cells, tumor vaccines and other immunotherapy strategies have continuously emerged, which has led to rapid development of tumor immunotherapy. In theory, when tumor cells occur accidentally in the body, they will be recognized and cleared by the immune system, thereby maintaining the normal physiological activities of the body. However, in actual situations, the antigen expressed by tumor cells is mutated or not expressed, so that some tumor cells can escape from this equilibrium state, and further develop and metastasize, that is, immune escape occurs 1,2. In 2002, Schreiber and others first proposed the theory of tumor immune editing, which believed that tumor cells can go through the three stages of immune elimination, stalemate and escape, and finally escape the monitoring of the immune system 3.

Currently, immune checkpoint inhibitors (ICIs) have made significant progress in the treatment of various cancers. The immune checkpoint pathway is regulated by the interaction of ligands and receptors, so proteins related to this can become potential targets for ICIs. The current research on ICIs mainly focuses on CTLA-4 (cytotoxic T lymphocyte associated antigen-4) antibody, PD-1 (programmed cell death protein 1) antibody and PD-L1 (programmed cell death-ligand 1) antibody. It has been gradually approved for the treatment of patients with melanoma, non-small cell lung cancer (NSCLC), classical Hodgkin lymphoma (cHL), head and neck squamous cell carcinoma (HNSCC), and urothcarcinoma (RCCa) 4.

However, due to the high heterogeneity of tumors, and many inhibitory immune checkpoint molecules jointly promote the immune escape of tumor cells, the use of a single target immunotherapy strategy may leave a lot of space for tumor survival. At the same time, due to the different patients with different ICI reaction rate, make the broad spectrum of ICI has been challenged. ICI two-drug combination therapy can make up for the shortcomings of monotherapy. When the drug appears immune tolerance, it can play a synergistic anti-tumor effect by interfering with other targets, so ICI two-drug combination therapy may become a development direction of tumor immunotherapy in the future. Moreover, for patients with chemotherapy contraindications, the combination of two ICI therapy has more advantages than chemotherapy combined with ICI therapy. At present, ICI two-drug combination therapy has shown a certain effect in various cancers such as melanoma, lung cancer, gastric cancer and mesothelioma. However, the number of large-scale clinical trials is currently small, and the toxic and side effects caused by ICI by promoting T cell activation and autoimmune reactions are still controversial. We performed a meta-analysis to analyze the efficacy and safety of anti-CTLA-4 combined with anti-PD-1/PD-L1 and ICI monotherapy in patients with advanced cancer.

## Material and methods

### Search strategy

**Figure 1:**
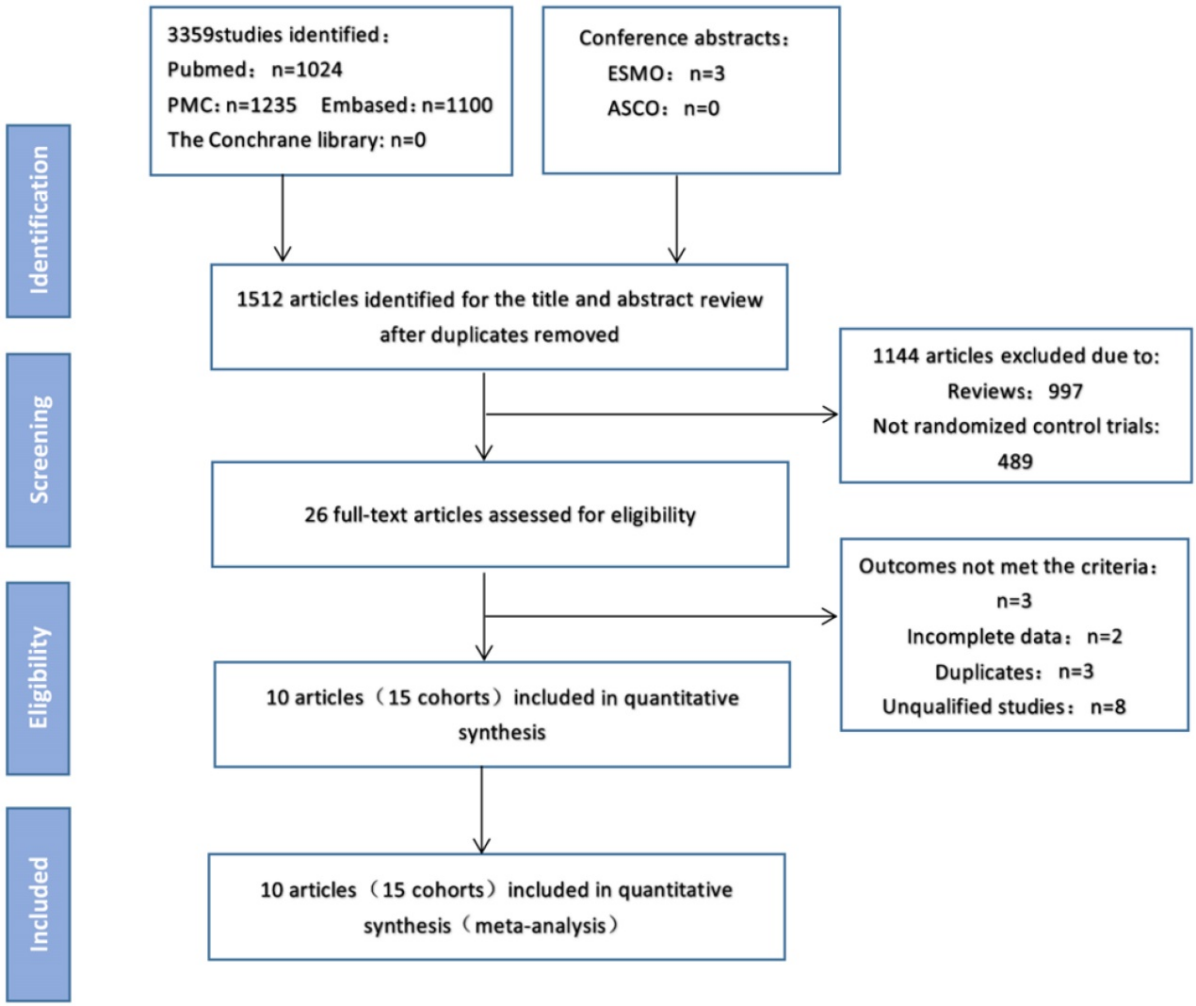
Study flow diagram.

As of April 15, 2020, electronic searches were performed on PubMed, PMC, MEDLINE, EMBASE, Cochrane Central Register of Controlled Trials (CENTRAL), American Society of Clinical Oncology (ASCO), European Medical Oncology (ESMO), http://www.clinicaltrials.gov/. The detailed search strategy is shown in Figure 1.The search term was as follows: (“Nivolumab” OR “Pembrolizumab” OR “Atezolizumab” OR “Ipilimumab” OR “Tremelimumab” OR “Durvalumab” OR “Lambrolizumab” OR “Avelumab” OR “immune checkpoint inhibitors” OR “immune checkpoint inhibitor” OR “immuno checkpoint inhibitors” OR “immuno checkpoint inhibitor” OR “ICI” OR “ICIs” ) AND (“Neoplasia” OR “Neoplasias” OR “Neoplasm” OR “Tumors” OR “Tumor” OR “Cancer” OR “Cancers” OR “Malignancy” OR “Malignancies”) AND (“randomized controlled trial” OR “randomized” OR “placebo”). We searched all potentially relevant studies and reviewed the references in the final included articles to find possible missing studies.

### Inclusion criteria

The included studies should meet the following inclusion criteria: (1) Advanced esophageal and gastric junction cancer/stomach cancer, lung cancer, melanoma, head and neck squamous cell carcinoma diagnosed by cytology or histology pathology; (2) Randomized controlled trial (RCT) comparing ICI combined with chemotherapy and chemotherapy alone; (3) Outcome indicators included the number of people who achieved objective response rate (ORR) and disease control rate (DCR), and the hazard ratio (HR) of progression-free survival (PFS) and OS, as well as their 95% confidence interval (95% CI), between the experimental group and the control group. (4) The number of people achieving ORR was the sum of complete response (CR) and partial response (PR), and the number of people achieving DCR was the sum of CR, PR and stable disease (SD).

### Data extraction

Two independent researchers extracted data from the included studies based on the preferred report project (PRISMA) for systematic evaluation and meta-analysis. All inconsistencies were resolved with the unanimous consent of all researchers. Information collected from these studies includes title, first author, year of publication, number of patients, median age, treatment plan, medication dose, number of people achieving ORR and DCR, HR and 95% CI of PFS and OS.

### Quality assessment

The Cochrane collaboration tool was used to assess the risk of bias for each study. This included the following assessment scopes: random sequence generation, allocation hiding, blindness of participants and researchers, blindness of outcome evaluators, incomplete outcome data, selective reporting, and other biases. According to the matching degree between the extracted information and the evaluation criteria, the risks in each field were classified as high risk, uncertain risk or low risk. GRADE was used to assess the level of evidence for all analysis results, which were classified as high quality, medium quality, low quality and very low quality.

### Statistical analysis

Statistical analysis was performed using Review Manager 5.3 and Forest plots were made. The main end point of the meta-analysis was to compare the efficacy of ICI combination therapy and ICI monotherapy alone, and the evaluation indicators were risk ratio (RR) and its 95% CI of ORR and DCR, and HR and its 95% CI of PFS and OS. State 12.0 was used to evaluate publication bias based on Begg's and Egger's tests. Heterogeneity between studies was represented by Cochrane's X^2^ statistics and the inconsistency statistic (I^2^). We considered I^2^ <50% as low-level heterogeneity and I^2^ > 50% as significant heterogeneity. When I^2^ < 50%, the fixed effect model was used. When I^2^ > 50%, the random effects model was used. In addition, we used sensitivity analysis to test the stability of the results. In order to further explore the efficacy of ICI combination therapy, subgroup analysis was performed by line of therapy, ICI drugs in the control group, and tumor type. In all included studies, *p* < 0.05 was considered statistically significant.

## Results

### Characteristics of the included studies

Figure 1 shows the flow chart of this study. A total of 3362 records were retrieved through a database search. 1512 articles remaining after excluding duplicates, of which 138 were eliminated after reading the titles and abstracts. After excluding articles that did not meet the requirements, the full text of the remaining 26 articles was then reviewed, and 10 studies (15 cohorts) 5,6,7,8,9,10,11,12,13,14 involving 2410 patients were finally included in the meta-analysis. In these studies, the sample size of the included trials ranged from 19 to 945. Of these, three were phase III trials, seven were phase I/II trials, and three were first-line treatments, seven were second and above line treatments. Table 1 and sTable 1 summarize the characteristics of the included studies.

### Quality assessment of included studies

**Figure 2:**
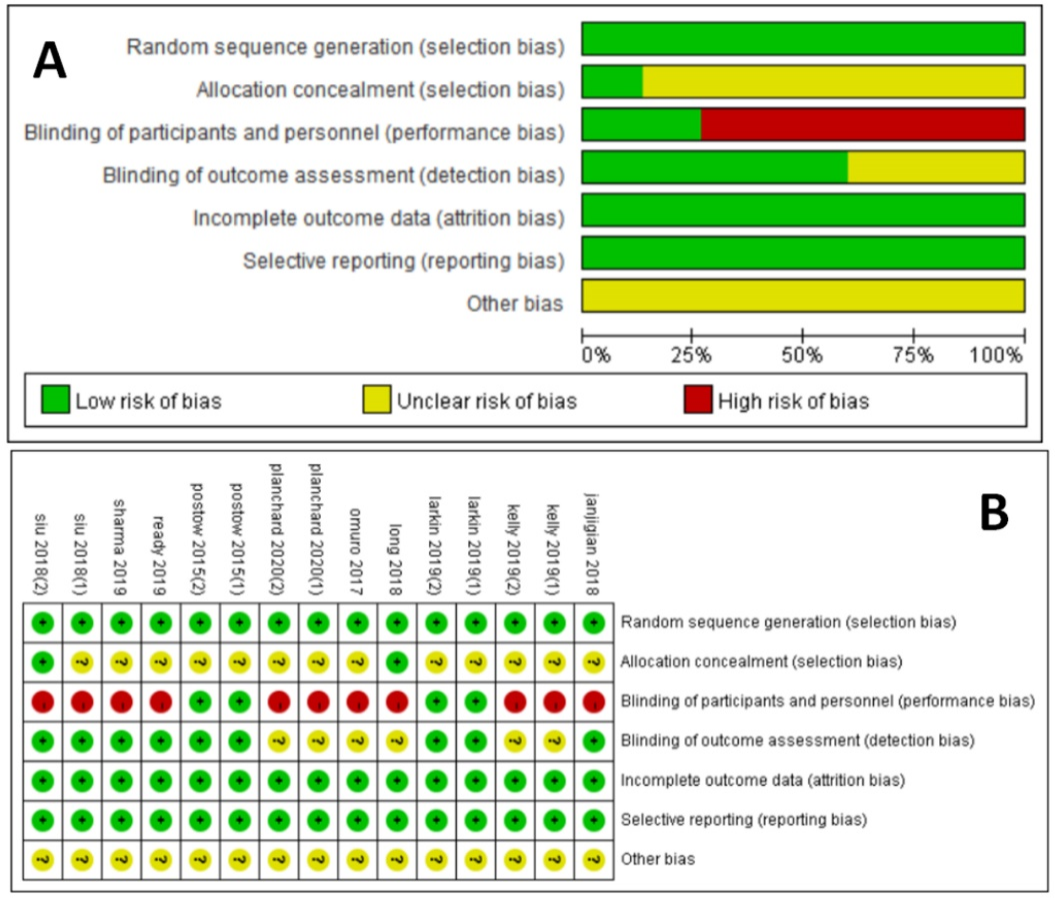
Assessment of risk of bias. (A) Risk of bias summary. (B) Risk of bias graph.

We critically evaluated the methodological quality of the included studies based on the Cochrane Collaborative Bias Risk Tool. Because of the authors' detailed description of the randomization principle, all included trials were rated as having a low risk of randomization bias. Other sources of bias were not identified. The graphical results of methodological quality are shown in Figure 2. The risk of bias items for each included study is presented in Figure 2.

### Overall response rate (ORR) and disease control rate (DCR)

**Figure 3:**
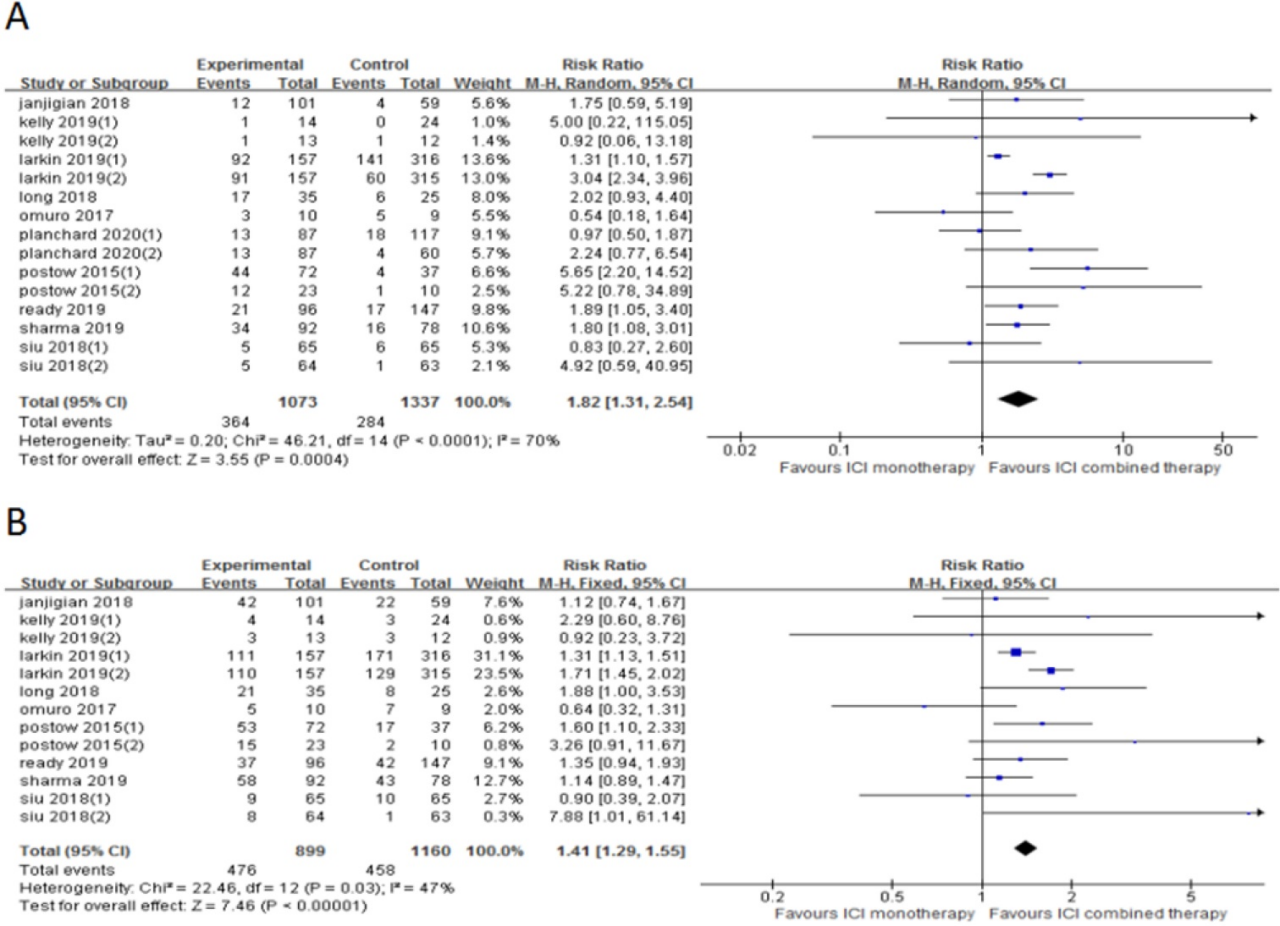
Forest plot and pooled RR and 95% CI for ORR (A) and DCR (B): “ICI combination therapy” versus “ICI monotherapy”. RR, relative ratio; CI, confidence interval; ORR, objective response rate; DCR, objective response rate; ICI, immune checkpoint inhibitor.

**Figure 4:**
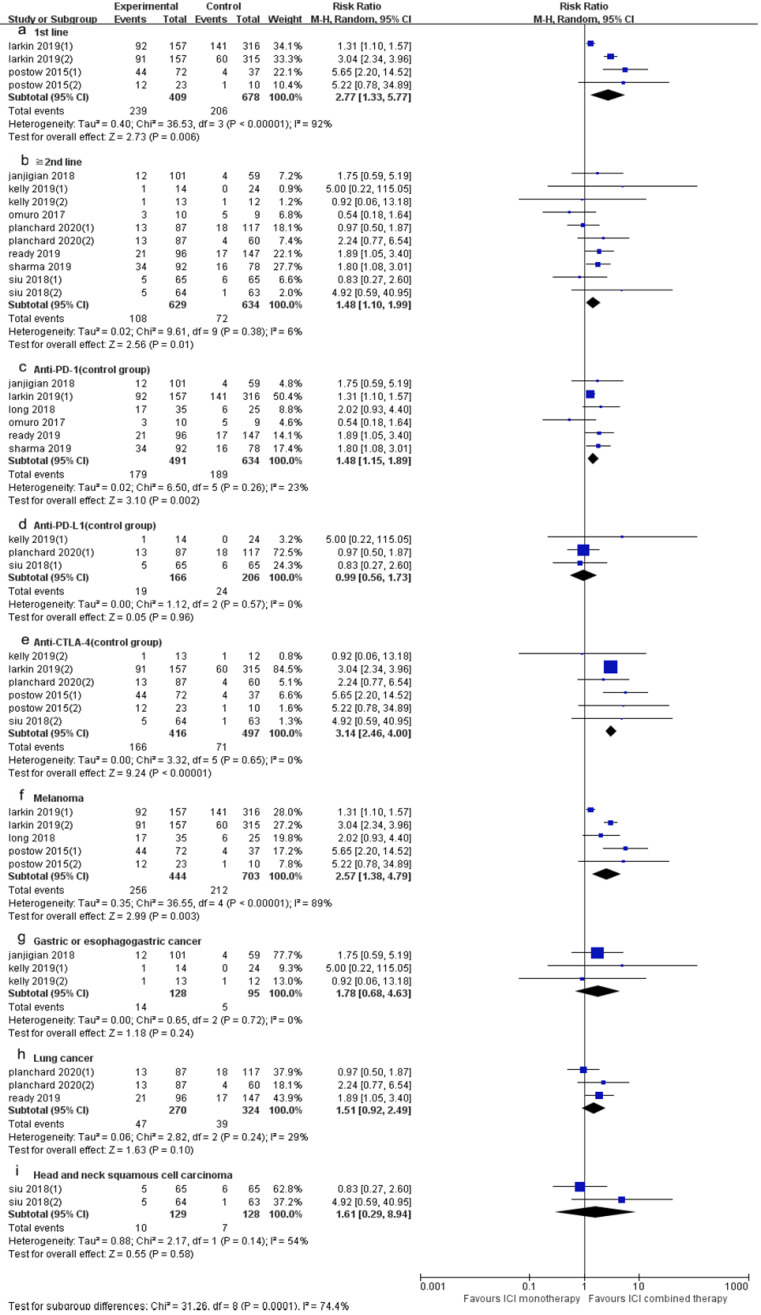
Forest plot and pooled RR and 95% CI for subgroup ORR: “ICI combination therapy” versus “ICI monotherapy”. (a: ORR of 1st line; b: ORR of “≥2^nd^ line”; c: ORR of anti-PD-1 therapy in control group; d: ORR of anti-PD-L1 therapy in control group; e: ORR of anti-CTLA4 therapy in control group; f: ORR of subgroup of melanoma; g: ORR of gastric or esophagogastric cancer; h: ORR of lung cancer; i: ORR of head and neck squamous cell carcinoma) RR, risk ratios; CI, confidence intervals; ORR, objective response rate; ICI, immune checkpoint inhibitor.

10 trials (15 cohorts) included reported ORR, and 9 trials (13 cohorts) reported DCR. The ORR range of the ICI combination therapy group was 7-61%, and the DCR range was 13-74%. The combined data showed that ORR was 34% and 21% in ICI combination therapy group and ICI monotherapy group, respectively, and DCR was 53% and 39% in ICI combination therapy group and ICI monotherapy group, respectively. The ORR of ICI combination therapy was significantly higher than that of ICI monotherapy (RR 1.82, 95% CI 1.31-2.54, *p* = 0.0004, Fig. 3A), and the heterogeneity was greater (*p* <0.0001, I^2^ = 70%). Therefore, we did a sensitivity analysis. After excluding the Kelly's study with the smallest weight (1.0%), there was no significant change in heterogeneity. After excluding the Larkin's study with the largest weight (13.6%), the heterogeneity decreased (I^2^ = 53%, *p* = 0.0002), but the statistical results were relatively stable and unchanged. To further explore the sources of heterogeneity, we conducted subgroup analysis based on line of therapy, ICI drugs in the control group, and tumor type (Fig. 4). The results showed that in 1st line (RR 2.77, 95% CI 1.33-5.77, *p* = 0.006, Fig. 4a), “≥ 2^nd^ line” (RR 1.48, 95% CI 1.10-1.99, *p* = 0.01, Fig. 4b), anti-PD-1 (control group) (RR 1.48, 95% CI 1.15-1.89, *p* = 0.002, Fig. 4c), anti-CTLA-4 (control group) (RR 3.14, 95% CI 2.46-4.00, *p* < 0.00001, Fig. 4e) and melanoma (RR 2.57, 95% CI 1.38-4.79, *p* = 0.003, Fig. 4f) groups, the ORR of ICI combination therapy was significantly higher than that of ICI monotherapy. In anti-PD-L1 (control group) (RR 0.99, 95% CI 0.56-1.73,* p* = 0.96, Fig. 4d), gastric or esophagogastric cancer (RR 1.78, 95% CI 0.68-4.63,* p* = 0.24, Fig. 4g), lung cancer (RR 1.51, 95% CI 0.92-2.49, *p* = 0.10, Fig. 4h) and head and neck squamous cell carcinoma (RR 1.61, 95% CI 0.29-8.94, *p* = 0.58, Fig. 4i) groups, there was no statistically significant difference in ORR between ICI combination therapy and ICI monotherapy. According to the subgroup analysis, we found great heterogeneity in 1st line (*p* < 0.00001, I^2^ = 92%) and melanoma (*p* < 0.00001, I^2^ = 89%) groups. Heterogeneity of head and neck squamous cell carcinoma (*p* =0.08, I^2^ = 51%) group was relatively large. Other groups showed less heterogeneity.

**Figure 5:**
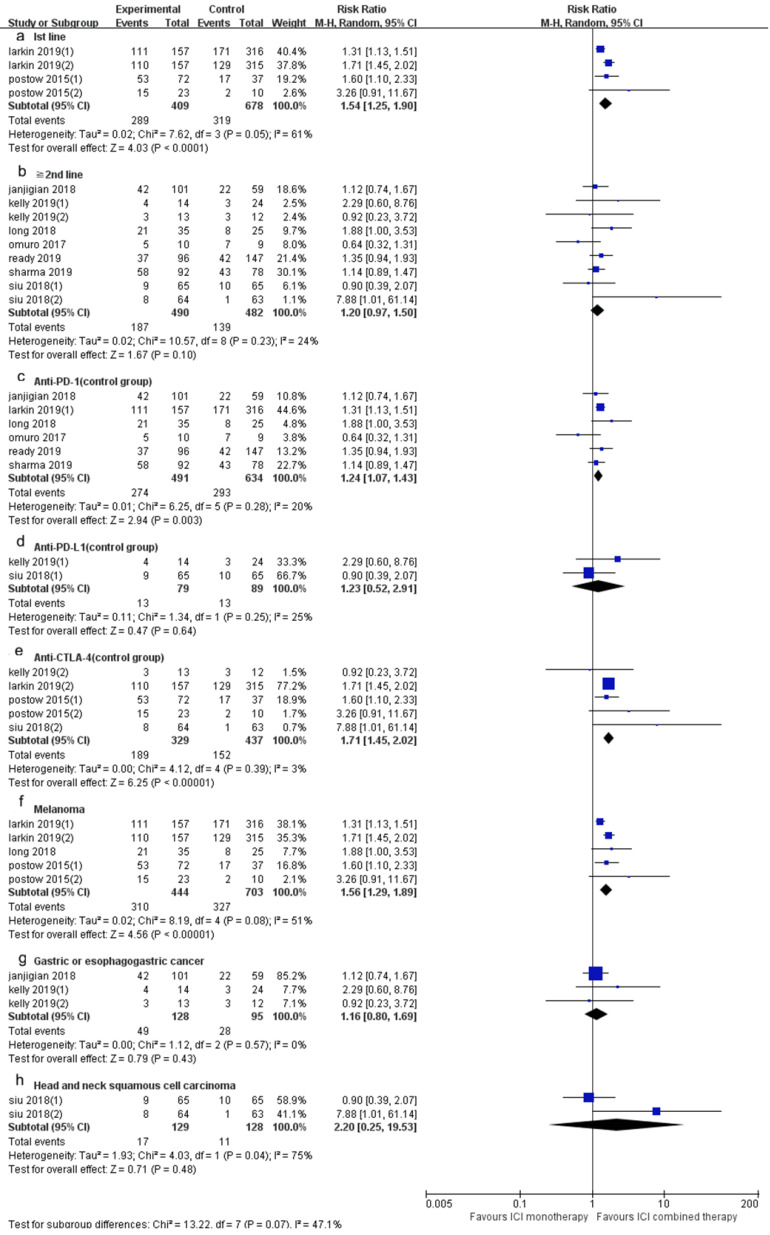
Forest plot and pooled RR and 95% CI for subgroup DCR: “ICI combination therapy” versus “ICI monotherapy”. (a: DCR of 1st line; b: DCR of “≥2^nd^ line”; c: DCR of anti-PD-1 therapy in control group; d: DCR of anti-PD-L1 therapy in control group; e: DCR of anti-CTLA4 therapy in control group; f: DCR of melanoma; g: DCR of gastric or esophagogastric cancer; h: DCR of head and neck squamous cell carcinoma) RR, risk ratios; CI, confidence intervals; DCR, objective response rate; ICI, immune checkpoint inhibitor.

The DCR of ICI combination therapy was significantly higher than that of ICI monotherapy (RR 1.41, 95% CI 1.29-1.55, *p* < 0.0001, Fig. 3B), and there was heterogeneity (*p* = 0.03, I^2^ = 47%). Sensitivity analysis indicated that after excluding the Siu's study with the smallest weight (0.3%) and the Larkin's study with the largest weight (31.1%), there was no significant change in heterogeneity and statistical results. A subgroup analysis (Fig. 5) was also conducted, and the results showed that in 1st line (RR 1.54, 95% CI 1.25-1.90, *p* < 0.0001, Fig. 5a), anti-PD-1 (control group) (RR 1.24, 95% CI 1.07-1.43, *p* = 0.003, Fig. 5c), anti-CTLA-4 (control group) (RR 1.71, 95% CI 1.45-2.02, *p* < 0.00001, Fig. 5e) and melanoma (RR 1.56, 95% CI 1.29-1.89, *p* < 0.00001, Fig. 5f) groups, the DCR of ICI combination therapy was significantly higher than that of ICI monotherapy. In “≥ 2^nd^ line” (RR 1.20, 95% CI 0.97-1.50, *p* = 0.10, Fig. 5b), anti-PD-L1 (control group) (RR 1.23, 95% CI 0.52-2.91, *p* = 0.64, Fig. 5d), gastric or esophagogastric cancer (RR 1.16, 95% CI 0.80-1.69, *p* = 0.43, Fig. 4g) and head and neck squamous cell carcinoma (RR 2.20, 95% CI 0.25-19.53, *p* = 0.48, Fig. 5h), there was no significant change in DCR of ICI combination therapy and ICI monotherapy. According to the subgroup analysis, we found great heterogeneity in 1^st^ line (*p* = 0.05, I^2^ = 61%) and head and neck squamous cell carcinoma (*p* = 0.04, I^2^ = 75%) groups. Heterogeneity of melanoma (*p* = 0.08, I^2^ = 51%) group was relatively large. Other groups showed less heterogeneity.

### Progression-free survival (PFS) and overall survival (OS)

**Figure 6:**
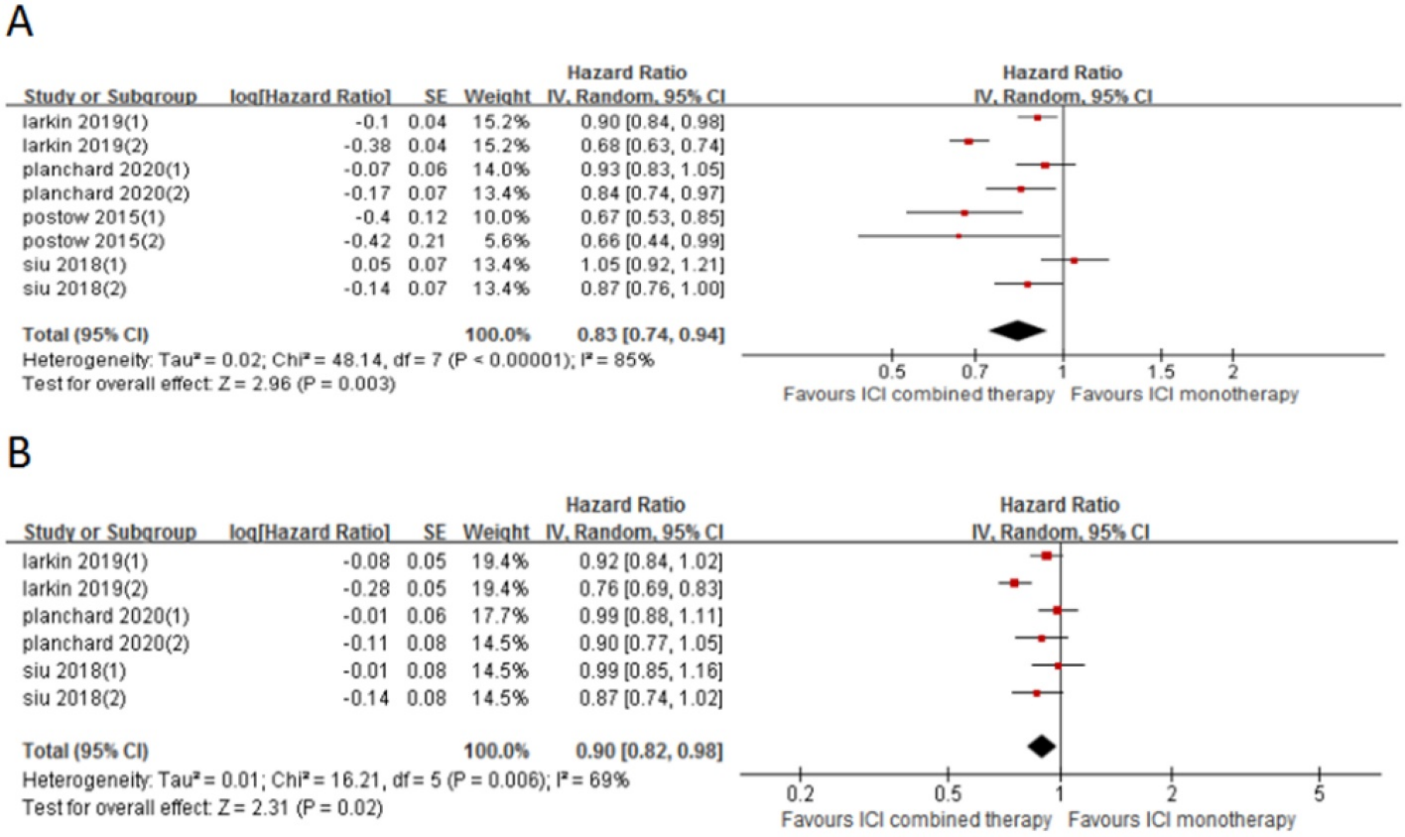
Forest plot and pooled HR and 95% CI for PFS (A) and OS (B): “ICI combination therapy” versus “ICI monotherapy”. HR, hazard ratio; CI, confidence interval; PFS, progression free survival; OS, progression free survival; ICI, immune checkpoint inhibitor.

**Figure 7:**
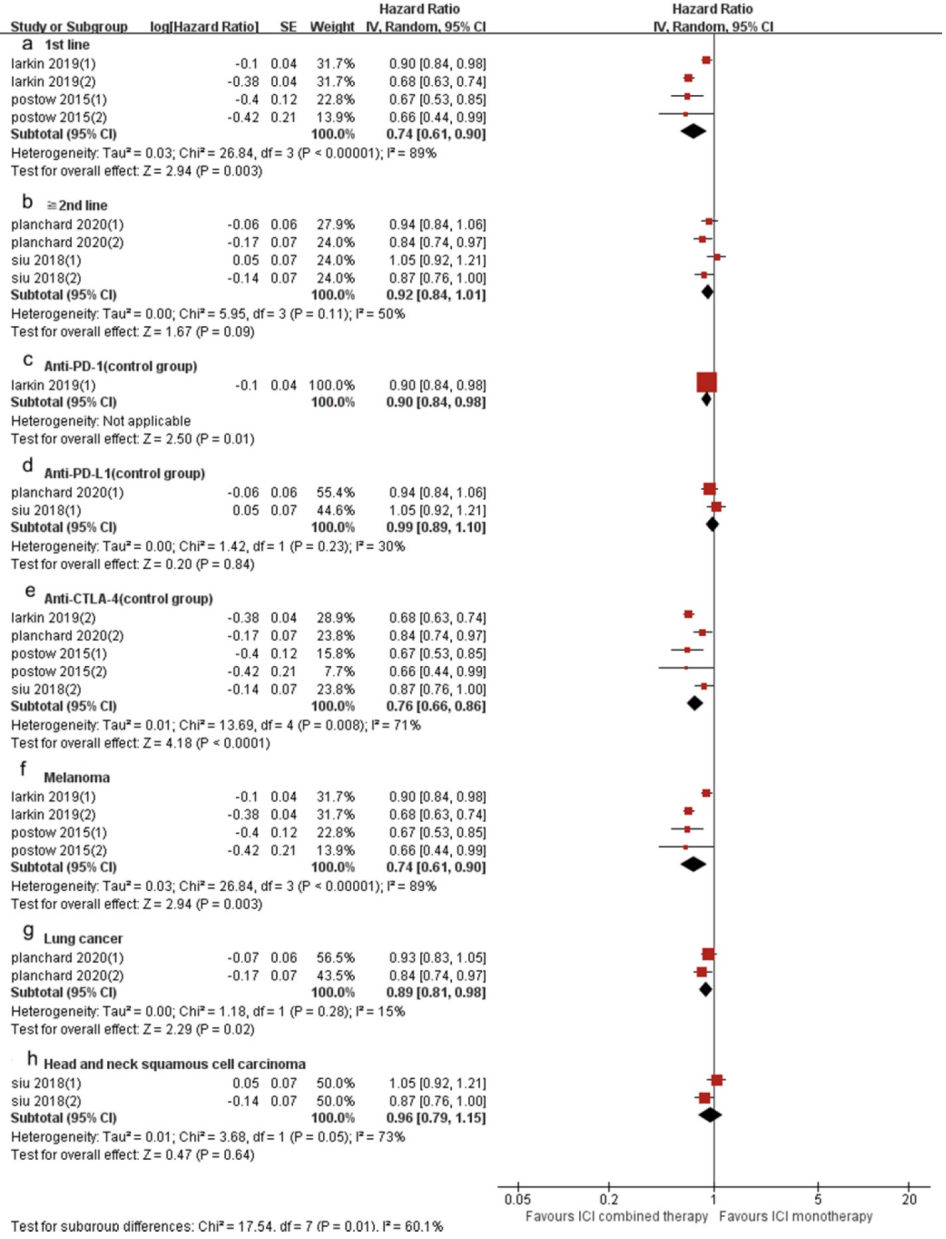
Forest plot and pooled HR and 95% CI for subgroup PFS: “ICI combination therapy” versus “ICI monotherapy”. (a: PFS of 1st line; b: PFS of “≥2^nd^ line”; c: PFS of anti-PD-1 therapy in control group; d: PFS of anti-PD-L1 therapy in control group; e: PFS of anti-CTLA4 therapy in control group; f: PFS of melanoma; g: PFS of lung cancer; h: PFS of head and neck squamous cell carcinoma) HR, hazard ratios; CI, confidence intervals; PFS, progression free survival; ICI, immune checkpoint inhibitor.

4 included trials (8 cohorts) reported PFS, and 3 trials (6 cohorts) reported OS. The combined results showed that ICI combination therapy has significantly longer PFS than ICI monotherapy (HR 0.83, 95% CI 0.74-0.94, *p* = 0.003, Fig. 6A), and the heterogeneity was greater (*p* < 0.0001, I^2^ = 85%). Therefore, we did a sensitivity analysis. After excluding the Postow's study with the smallest weight (5.6%) and the Larkin's study with the largest weight (15.1%), there was no significant change in heterogeneity and statistical results. To further explore the sources of heterogeneity, we conducted a subgroup analysis (Fig. 7). The results show that in 1st line (HR 0.74, 95% CI 0.61-0.90, *p* = 0.003, Fig. 7a), anti-PD-1 (control group) (HR 0.90, 95% CI 0.84-0.98, *p* = 0.01, Fig. 7c), anti-CTLA-4 (control group) (HR 0.76, 95% CI 0.66-0.86,* p* < 0.0001, Fig. 7e), melanoma (HR 0.74, 95% CI 0.61-0.90, *p* = 0.003, Fig. 7f) and lung cancer (HR 0.89, 95% CI 0.81-0.98, *p* = 0.02, Fig. 7g) groups, the PFS of ICI combination therapy was significantly longer than that of ICI monotherapy. In “≥2^nd^ line” (HR 0.92, 95% CI 0.84-1.01, *p* = 0.09, Fig. 7b), anti-PD-L1 (control group) (HR 0.99, 95% CI 0.89-1.10, *p* = 0.84, Fig. 7d) and head and neck squamous cell carcinoma (HR 0.96, 95% CI 0.79-1.15, *p* = 0.64, Fig. 7h) groups, there was no significant difference in PFS between ICI combination therapy and ICI monotherapy. According to the subgroup analysis, we found that great heterogeneity in 1st line (*p* < 0.00001, I^2^ = 89%), anti-CTLA-4 (control group) (*p* = 0.008, I^2^ = 71%), melanoma (*p* < 0.00001, I^2^ = 89%), head and neck squamous cell carcinoma (*p* =0.05, I^2^ = 73%) groups. Other groups showed less heterogeneity.

**Figure 8:**
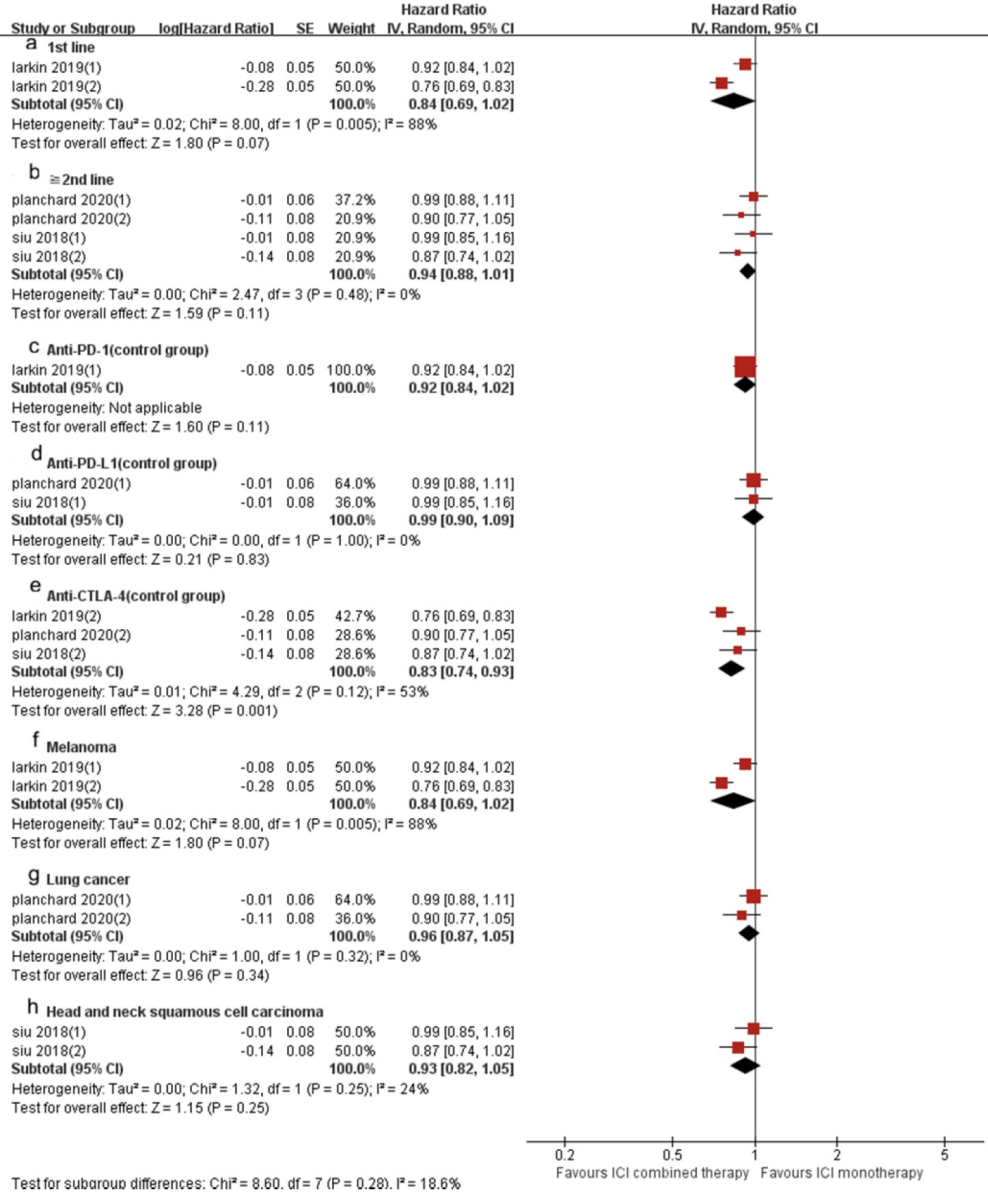
Forest plot and pooled HR and 95% CI for subgroup OS: “ICI combination therapy” versus “ICI monotherapy”. (a: OS of 1st line; b: OS of “≧2nd line”; c: OS of anti-PD-1 therapy in control group; d: OS of anti-PD-L1 therapy in control group; e: OS of anti-CTLA4 therapy in control group; f: OS of melanoma; g: OS of lung cancer; h: OS of head and neck squamous cell carcinoma) HR, hazard ratios; CI, confidence intervals; OS, progression free survival; ICI, immune checkpoint inhibitor.

Similarly, the OS of ICI combination therapy was significantly longer than that of ICI monotherapy (HR 0.90, 95%CI 0.82-0.98, *p* = 0.02, Fig. 6B), and the heterogeneity was greater (*p* = 0.006, I^2^ = 69%). Sensitivity analysis showed that after excluding the Siu's study or Phanchard's study with the smallest weight (15.4%), there was no significant change in heterogeneity and statistical results. After excluding the Larkin's study with the largest weight (19.4%), the heterogeneity disappeared (*p* = 0.63, I^2^ = 0%). A subgroup analysis (Fig. 8) was also conducted. And the results showed that in anti-CTLA-4 (control group) (HR 0.83, 95%CI 0.74-0.93, *p* = 0.001, Fig. 8e) group, the PFS of ICI combination therapy was significantly prolonged compared with that of ICI monotherapy. In 1st line (HR 0.84, 95% CI 0.6-1.02, *p* = 0.07, Fig. 8a), “≥2^nd^ line” (HR 0.94, 95% CI 0.88-1.01, *p* = 0.11, Fig. 8b), anti-PD-1 (control group ) (HR 0.92, 95% CI 0.84-1.02, *p* = 0.21, Fig. 8c), anti-PD-L1 (control group) (HR 0.99, 95% CI 0.90-1.09, *p* = 0.83, Fig. 8d), melanoma (HR 0.74 , 95% CI 0.61-0.90, *p* = 0.003, Fig. 8f), lung cancer (HR 0.96, 95% CI 0.87-1.05, *p* = 0.34, Fig. 8g) and head and neck squamous cell carcinoma (HR 0.93, 95% CI 0.82-1.05, *p* = 0.25, Fig. 8h) groups, there was no significant difference in OS between ICI combination therapy and ICI monotherapy. According to the subgroup analysis, we found great heterogeneity in 1st line (*p* = 0.005, I^2^ = 88%) and melanoma (*p* = 0.005, I^2^ = 88%) groups. In the anti-CTLA-4 (control group) (*p* = 0.12, I^2^ = 53%) group, the heterogeneity was relatively large. Other groups showed less heterogeneity.

### Safety outcomes

**Figure 9:**
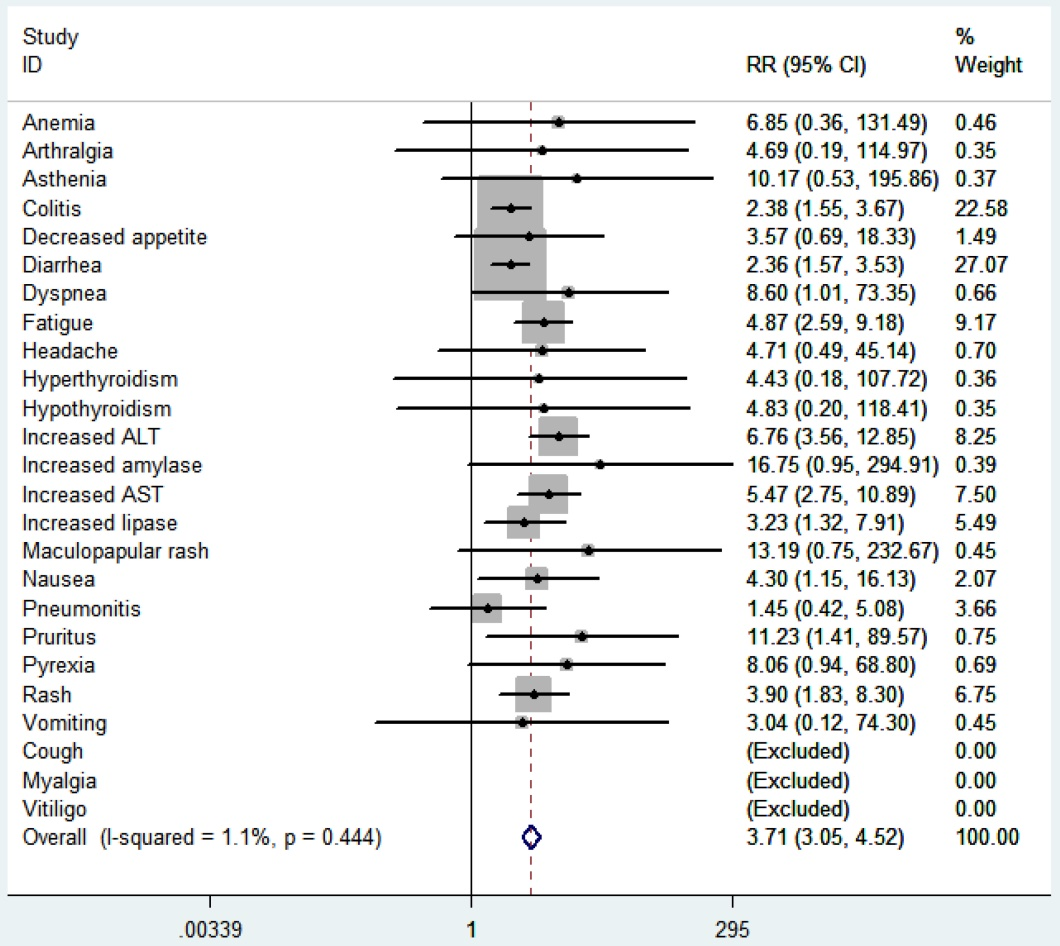
RR of high grade adverse events in cancer patients with treated with ICI combination. RR, risk ratios; ICI, immune checkpoint inhibitor.

According to the common toxicity criteria of the National Cancer Institute, the side effects (≥3) included in the study were evaluated (Fig. 9). In general, skin toxicity, gastrointestinal toxicity, liver toxicity, and lung toxicity are more common among patients treated with ICI. Among patients treated with ICI combination therapy, fatigue (RR 5.11, 95% CI 2.68-9.76, *p* < 0.00001), nausea (RR 4.35, 95% CI 1.15-16.48, *p* = 0.03), diarrhea (RR 2.48, 95% CI 1.62-3.79, *p* < 0.0001), colitis (RR2.52, 95% CI 1.59-3.98, *p* < 0.0001), rash (RR 4.02, 95% CI 1.86-8.66, *p* = 0.0004), pruritus (RR 11.36 , 95% CI 1.42-91.03, *p* = 0.02), elevated ALT (RR 7.28, 95% CI 3.77-14.04, *p* < 0.00001), elevated AST (RR 5.76, 95% CI 2.85-11.63, *p* < 0.00001), elevated lipase (RR 3.44, 95% CI 1.36-8.74, *p* = 0.009) were higher than ICI monotherapy. We further compared the safety of anti-CTLA-4 combined with anti-PD-1 or anti-PD-L1 (Table 2). The results showed that the side effects of anti-CTLA-4 combined with anti-PD-1 was higher than that of monotherapy (*p* < 0.00001), including fatigue (*p* < 0.00001), nausea (*p* = 0.02), diarrhea (*p* < 0.0001), colitis (*p* < 0.0001), rash (*p* = 0.0004), elevated ALT (*p* < 0.00001), elevated AST (*p* < 0.00001), elevated lipase (*p* = 0.01) significantly increased, but no significant difference between the side effects of anti-CTLA-4 combined with anti-PD-L1 and monotherapy (*p* = 0.34).

### Publication bias

Evaluation was performed by Egger's test and Begg's test. In Begg's test, z = 0.40 (continuity corrected), Pr > | z | = 0.692. In Egger's test, t = 0.53, *p* = 0.602. The results showed no publication bias.

### Evidence level

**Figure 10:**
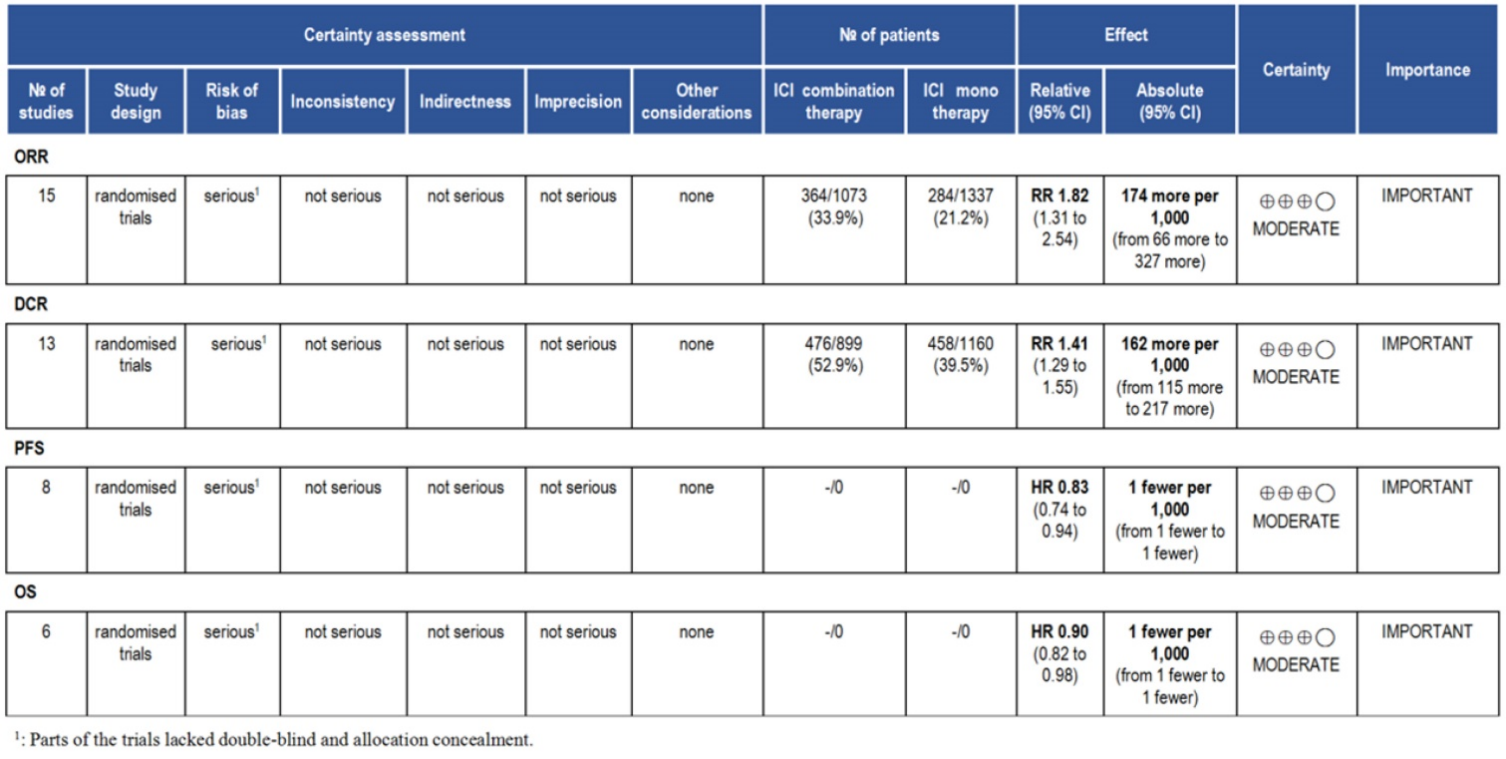
Summary of GRADE on evidences of outcomes.

According to the GRADE, we analyzed the evidence level of the results, and the results showed that the evidence level of ORR, DCR, PFS and OS were moderate (Fig. 10).

## Discussion

ICI is a type of therapy method that can improve the anti-tumor immune response by regulating the activity of T cells. Commonly referred to as the immune checkpoint is actually an inhibitory pathway in the immune system, their initial role is to avoid excessive immunity, but in cancer patients immune checkpoint is closely related to the immune escape of the tumor. Combined immunotherapy is the current treatment trend, including chemotherapy combined with ICI therapy and two combined ICI therapy. For patients with chemotherapy contraindications, the ICI two-drug combination therapy has more advantages than chemotherapy combined with ICI therapy. Existing clinical trials have shown that the ICI two-drug combination therapy has certain survival benefits 15,16. The results of this meta-analysis showed that the ORR, DCR, PFS and OS of anti-CTLA-4 combined with anti-PD-1/PD-L1 were significantly better than that of ICI monotherapy.

Some clinical trials have been published in recent years. The CheckMate 227 trial included 793 untreated stage IV or relapsed NSCLC patients. The results showed that the ORR (45.3%) of nivolumab combined with ipilimumab in first-line treatment of NSCLC was higher than that of chemotherapy alone (26.9%), and the mPFS of nivolumab combined with ipilimumab and chemotherapy alone was 7.2 months versus 5.5 months (*p* < 0.001), mOS was 17.1 months versus 14.9 months (*p* = 0.007), and was independent of the expression level of PD-L1 17. The CheckMate 204 trial included 94 untreated patients with advanced melanoma brain metastases. The results showed that the intracranial clinical benefit rate was 57% and the extracranial clinical benefit rate was 56% 18. In the non-contrast MAPS-II trial, patients with melanoma or NSCLC were randomly divided into nivolumab monotherapy group and nivolumab combined with ipilimumab treatment group. The results showed that both groups had clinical benefits, with DCR of 40% and 52%, and ORR of 19% and 28%, respectively, and the mPFS of nivolumab combined with ipilimumab treatment group was better than that of nivolumab monotherapy group (5.6 months versus 4.0 months) 19. This meta-analysis included 10 randomized controlled studies with a total of 15 cohorts. The results of the analysis showed that the ORR, DCR, PFS and OS of ICI combination therapy were significantly better than that of ICI monotherapy. The sensitivity analysis of ORR, DCR and PFS all indicated that the statistical results were relatively stable. In the sensitivity analysis of OS, the heterogeneity disappeared after the exclusion of Larkin's study. In order to further explore heterogeneity, we performed subgroup analysis, which was divided into groups from line of therapy, ICI drugs in the control group and tumor type. We found that in 1st line and melanoma groups, the ORR, DCR and PFS of ICI combination therapy were superior to ICI monotherapy, but there was no significant difference in OS. In “≥2^nd^ line” group, the ORR of ICI combination therapy was superior to ICI monotherapy, but there were no difference in those of DCR, PFS and OS. In the anti-PD-1 (control group) group, ORR, DCR and PFS of ICI combination therapy were superior to ICI monotherapy, but there was no difference in OS. In anti-PD-L1 (control group) group, there was no difference in ICI combination therapy and ICI monotherapy of ORR, DCR, PFS and OS. In anti-CTLA-4 (control group) group, ORR, DCR, PFS and OS of ICI combination therapy were superior to ICI monotherapy. In lung cancer group, the PFS of ICI combination therapy was superior to ICI monotherapy, but there were no significant difference in those of ORR and OS. In gastric or esophagogastric group, there were no significant difference in ICI combination therapy and ICI monotherapy of ORR and DCR. In head and neck squamous cell cancer group, there were no significant difference in ICI combination therapy and ICI monotherapy of PFS and OS.

In recent years, the research of ICI drugs is more and more extensive. CTLA-4 (CD-125) has a high homology with the co-stimulus receptor CD28 on the surface of T cells. It is also expressed on the surface of T cells and is a negative regulator of T cell activity. It can competitively bind to CD80/CD86 and block CD28/B7 costimulatory signals, thereby inhibiting the immune activity of T cells. PD-1 (CD-279), a member of the CD28 family, is also mainly expressed on the surface of T cells, and can also be expressed on the surface of B cells and monocytes. It is an inhibitory receptor that, when activated by binding with its ligands PD-L1 (B7-H1 /CD274) and PD-L2 (B7-H2/CD273), can cause T cell incapacitation, exhaustion or death 20-22. Although both are negative signals for T cell activation, their location and timing are different. CTLA-4 is expressed on T cells, while PD-1 is more widely expressed on a variety of cells. Normally, CTLA-4 suppresses T cells in the early stages of the immune cycle in lymph nodes, while PD-1 regulates the immune response in peripheral tissues or tumor sites 21,23. At present, there have been a lot of reports on anti-CTLA-4, anti-PD-1 and anti-PD-L1. Studies 24 have shown that in the tumor microenvironment, anti-CTLA-4 stimulates the activation of surrounding T cells by blocking the binding of CTLA-4 on the surface of T cells to the ligand CD80/CD86 on APCs, but does not activate T cells. Anti-PD-1/PD-L1 may play an anti-tumor effect by activating T lymphocytes. Reck also believes that because anti-CTLA-4 only plays a role in the immune activation stage, and lacks subsequent immune effectors, it cannot effectively stimulate sufficient anti-tumor immune responses 25. Pardoll's study demonstrated that anti-PD-1 targeting tumor infiltrating lymphocytes (TIL) can complement the anti-tumor activity of anti-CTLA-4 through non-redundant pathways 26. Our meta-analysis showed that the ORR, DCR, PFS and OS of anti-CTLA-4 combined with anti-PD-1 / PD-L1 were significantly better than those of anti-CTLA-4 monotherapy, which also verified the above view. We also considered the difference between anti-PD-1 and anti-PD-L1. Anti-PD-1 is not only bound to PD-L1, but also to PD-L2. PD-L2 also belongs to the B7 family of ligands. In addition to being selectively expressed on some tumor cells, it is mainly expressed on APCs. It participates in the formation of its own peripheral immune tolerance by inhibiting T cell proliferation and cytokine production 27. Studies have shown that the affinity of PD-L2 and PD-1 is 3-4 times greater than PD-L1 28,29. Anti-PD-L1 only blocks the PD-1 ~ PD-L1 pathway and does not affect the PD-1 ~ PD-L2 pathway. Experiments have proved that the expression of PD-L2 in different tumors varies greatly. In melanoma tissue samples, only a few tumor cells expressed PD-L2; in gastric cancer, the expression of PD-L2 increased; while in more than half of the HNSCC samples expressed PD-L2 30. According to our subgroup analysis of tumor type, the conclusions were also consistent with the above views. In recent years, ICIs have gradually moved from second-line therapy to first-line therapy. In patients who have not received chemotherapy and other treatments, the immune cells in their bodies are in a relatively complete and active state, so that the combination therapy of anti-CTLA-4 and anti-PD-1/anti-PD-L1 can play a synergistic role on the two pathways 23. For patients who have received more than one line of chemotherapy, systemic chemotherapy is usually considered to be immunosuppressive. Some chemotherapy drugs may reduce the effect of immunotherapy by inducing immunogenic danger signals in dying cancer cells, thus stimulating protective anti-tumor immunity 31. In our subgroup analysis, the effect of ICI combination therapy in first-line therapy was more significant than that in second and above line therapy, which further validated the above views. Malignant melanoma is one of the most sensitive malignant tumors for immune regulation, and often shows good antigenicity and immunogenicity 31,32. Most patients with melanoma cells have relatively high numbers of mutations in their DNA due to ultraviolet radiation and melanobiology. These mutations lead to changes in the protein sequence that are easily recognized by T cell responses 33,34. Studies have shown that strong lymphocyte infiltration often occurs in the primary site of melanoma patients 35. Our subgroup analysis also confirmed that melanoma was more effective in ICI combination therapy than other solid tumors.

The main concern about the combined application of anti-CTLA-4 and anti-PD-1/PD-L1 is the safety and tolerability of treatment. Immune-related toxic and side effects are managed in accordance with safety guidelines, and most level 2-4 immune-related events can be effectively managed. In this meta-analysis, the side effects (≥ 3) in the ICI combination therapy group were higher than those in the ICI monotherapy group, in which fatigue, nausea, diarrhea, colitis, rash, pruritus, elevated transaminase and lipase were significantly increased. However, there was no significant difference in two groups about decreased appetite, vomiting, maculopapular rash, dyspnea, pneumonia, anemia, asthenia, arthralgia, headache, pyrexia, hyperthyroidism and hypothyroidism. Most side effects are easier to control, and no grade 5 toxicity has been observed. In the systematic evaluation of the safety of ICI combined treatment, Abdelhafeez 2 indicated that fatigue, nausea, diarrhea, rash, elevated AST and lipase in ICI combination therapy group was larger than that in ICI monotherapy group, while there was no significant difference in other side effects, which was basically consistent with our analysis results. In addition, the combination of lower doses of ipilimumab (1 mg/kg) and standard doses of nivolumab or pembrolizumab were shown to reduce the incidence of higher levels of toxicity in some single-arm studies and randomized phase II trials 36. But more research is needed to prove the effects of reduced doses. We further compared the safety of anti-CTLA-4 combined with anti-PD-1 or anti-PD-L1. The results showed that the side effects of anti-CTLA-4 combined with anti-PD-1 was higher than those of monotherapy (*p* < 0.00001), but there was no significant difference in side effects between anti-CTLA-4 combined with anti-PD-L1 and monotherapy (*p* = 0.34). This reminds us that while using anti-PD-L1 treatment, adding anti-CTLA-4 may not increase the toxic and side effects of the drug. In addition, studies have shown that PD-L1 not only binds to PD-1, but also binds to CD80, and PD-L1/CD80 interaction also transmits inhibitory signals in T cells 37,38. This suggests that anti-PD-L1 can improve immune effects through other pathways, which may provide a new idea for the therapeutic mechanism of anti-PD-L1 combined with anti-CTLA-4. However, due to the limited research we have included and the small number of cases of anti-PD-L1 combined with anti-CTLA-4 treatment, no positive conclusion has been drawn.

Finally, we analyzed the quality of the included studies and the level of evidence for the outcome indicators discussed. The inclusion of relatively high-quality studies suggests the reliability of the meta-analysis. GRADE indicated that the level of evidence for ORR, DCR, PFS and OS were moderate, and further clinical studies are needed to confirm it.

## Limitations

The current meta-analysis is limited by several aspects. First, we included 10 RCTs, but the sample size varied between studies, resulting in a smaller sample size among different subgroups, which may be the main source of affecting the quality of meta-analysis. Second, differences in patient statistical quality, follow-up time, course of treatment, and ethnicity lead to heterogeneity. Finally, this was an experimental meta-analysis based on data from studies rather than individual patients. We still lack patient's complications, ECOG score, disease progression and other factors affecting prognosis, which may play important roles in the efficacy of ICI combination therapy.

## Conclusions

ICI two-drug combination therapy was superior to ICI monotherapy in ORR, DCR, PFS and OS, but the side effects of ICI combination therapy were higher than ICI monotherapy and most of the side effects were easier to control. Further clinical trials are needed to confirm the effect of dose reduction on efficacy and adverse reactions in order to improve clinical efficacy and patient prognosis.

## Figures and Tables

**Table 1:** Characteristics of the included studies

Study	Region	Tumor	Phase	Line	Treatment arms	No. of patients	ORR (%)	DCR (%)	Progression-free survival	Overall survival
									HR (95% CI)	HR (95% CI)
Jianjigian 2018	North America, Europe	Esophagogastric cancer	1/2	2^nd^	Nivolumab+ipilimumab	101	12 (12)	42 (42)	Not given	Not given
					Nivolumab	59	4 (7)	22 (37)		
Kelly 2019	North America, Europe, Asia	Gastric/esophagogastric cancer	2	2^nd^	Durvalumab+tremelimumab	27	2 (7)	7 (26)	Not given	Not given
					Durvalumab	24	0 (0)	3 (1)		
					Tremelimumab	12	1 (8)	3 (25)		
Larkin 2019	South America, Europe, Asia, Africa	Melanoma	3	1^st^	Nivolumab+ipilimumab	314	183 (58)	221 (70)	A:0.79 (0.64-0.96)	A:0.83 (0.67-1.03)
					Nivolumab	316	141 (45)	171 (54)	B:0.42 (0.35-0.51)	B:0.52 (0.42-0.64)
					Ipilimumab	315	60 (19)	129 (41)		
Long 2018	Australia	Melanoma (brain metastases)	2	Not given	Nivolumab + ipilimumab	35	17 (49)	21 (60)	Not given	Not given
					Nivolumab	25	6 (24)	8 (32)		
Omuro 2017	North America	Glioblastoma	1	≥2^nd^	Nivolumab + ipilimumab	10	3 (30)	5 (50)	Not given	Not given
					Nivolumab	9	5 (56)	7 (78)		
Planchard 2020	North America, South America, Europe, Asia, Australia	NSCLC	3	≥3^rd^	Durvalumab+tremelimumab	174	25 (14)	Not given	A:0.87 (0.68-1.12)	A:0.98 (0.74-1.30)
					Durvalumab	117	18 (15)	Not given	B:0.67 (0.49-0.92)	B:0.78 (0.56-1.11)
					Tremelimumab	60	4 (7)	Not given		
Postow 2015	North America, Europe	Melanoma (BRAF-)	1	1^st^	Nivolumab + ipilimumab	72	44 (61)	53 (74)	0.40 (0.23-0.68)	Not given
					Ipilimumab	37	4 (11)	17 (46)		
		Melanoma (BRAF+)	1	1^st^	Nivolumab + ipilimumab	23	12 (52)	15 (65)	0.38 (0.15-1.00)	Not given
					Ipilimumab	10	1 (10)	2 (20)		
Ready 2019	North America, Europe	SCLC	1/2	≥2^nd^	Nivolumab + ipilimumab	96	21 (22)	37 (39)	Not given	Not given
					Nivolumab	147	17 (12)	42 (29)		
Sharma 2019	North America, Europe	Urothelial carcinoma	1/2	≥2^nd^	Nivolumab + ipilimumab	92	34 (37)	58 (63)	Not given	Not given
					Nivolumab	78	16 (21)	43 (55)		
Siu 2018	North America, Europe, Asia, Australia	HNSCC	2	2^nd^	Durvalumab+tremelimumab	129	16 (12)	17 (13)	A:1.13 (0.82-1.56)	A:0.99 (0.69-1.43)
					Durvalumab	65	6 (9)	10 (15)	B:0.73 (0.53-1.01)	B:0.72 (0.51-1.03)
					Tremelimumab	63	1 (2)	1 (2)		

**Table 2:** Toxicity comparison between anti-PD-1 and anti-PD-L1

Toxicity	Anti-PD-1 (*p* value)	Anti-PD-L1 (*p* value)
Anemia	-	0.20
Asthenia	0.12	0.20
Colitis	<0.0001	0.76
Decreased appetite	0.13	0.79
Diarrhea	<0.0001	0.75
Dyspnea	0.05	-
Fatigue	<0.00001	0.51
Hyperthyroidism	0.36	0.54
Hypothyroidism	0.09	-
Increased ALT	<0.00001	0.79
Increased amylase	0.05	0.54
Increased AST	<0.00001	0.28
Increased lipase	0.01	0.99
Nausea	0.03	0.79
Pruritus	0.02	-
Rash	0.0004	0.79
Vomiting	0.49	-
	<0.00001	0.34
